# A SLIC-DBSCAN Based Algorithm for Extracting Effective Sky Region from a Single Star Image

**DOI:** 10.3390/s21175786

**Published:** 2021-08-28

**Authors:** Chenguang Shi, Rui Zhang, Yong Yu, Xingzhe Sun, Xiaodong Lin

**Affiliations:** 1 Innovation Academy for Microsatellites of Chinese Academy of Sciences, Room 426, Building 4, 99 Haike Road, Shanghai 201203, China; icescg@mail.ustc.edu.cn (C.S.); yuy@microsate.com (Y.Y.); linxd@microsate.com (X.L.); 2 University of Chinese Academy of Sciences, Beijing 100049, China; sundasa@mail.ustc.edu.cn

**Keywords:** star tracker, star image processing, interference suppression, vision saliency

## Abstract

The star tracker is widely used for high-accuracy missions due to its high accuracy position high autonomy and low power consumption. On the other hand, the ability of interference suppression of the star tracker has always been a hot issue of concern. A SLIC-DBSCAN-based algorithm for extracting effective information from a single image with strong interference has been developed in this paper to remove interferences. Firstly, the restricted LC (luminance-based contrast) transformation is utilized to enhance the contrast between background noise and the large-area interference. Then, SLIC (the simple linear iterative clustering) algorithm is adopted to segment the saliency map and in this process, optimized parameters are harnessed. Finally, from these segments, features are extracted and superpixels with similar features are combined by using DBSCAN (density-based spatial clustering of applications with noise). The proposed algorithm is proved effective by successfully removing large-area interference and extracting star spots from the sky region of the real star image.

## 1. Introduction

The attitude determination system is crucial for a spacecraft to perform precise space missions [1,2,3,4,5,6]. Nowadays, with the upsurge of satellite constellations, the fast increase in artificial satellites has brought enormous influence on space exploration and astronomical observation experiments. The requirements for star trackers, especially ones with high accuracy and high dynamic performance, are increasing [7,8]. The star image is the only source of data for star tracker, while it is usually rendered useless by various interferences which can affect star spot extraction.

Noise of an active pixel sensor (APS) consists in a combination of dark current, photo shot noise, single point noise, Gaussian noise, read noise, fixed pattern noise and reset noise, etc. [7,9]. In addition to the background noise, large-area interferences in the field of view of star trackers can also affect the attitude output. The interferences mainly include the following: (1) Energetic protons emitted by the Van Allen inner radiation belt, cosmic rays and solar proton events, as well as the track of other spacecrafts. They produce linear interferences in the image plane due to relative velocity difference [10]. (2) Diffuse light of satellite facility or component [11] can enter the star tracker, causing regular-shaped interference. (3) In real night sky observation and calibration experiments, some interferences such as the thin clouds would inevitably enter the field of view of star tracker [12]. They can produce irregular-shaped interferences. Furthermore, under the requirements of low-cost and high-performance for future star trackers, urban real night star observation becomes possible. The biggest problem for urban stargazing is that there are many irregular large-area interferences in the field of view. All of these factors can cause malfunctions in the star tracker.

In traditional astronomical image processing research, vast works have already been devoted to the development of algorithms in order to remove interferences in astronomical images. The interference suppression algorithm based on superposition of multiple images can remove these interferences [13,14,15,16,17]. Methods based on point spread function (PSF) [18,19,20,21] function by detecting their peaked profile which is incompatible with the extended point spread function of sources imaged by the atmosphere. The multi-feature matching algorithms [22,23,24,25,26,27] extract multiple features and remove interferences by geometric matching. It would increase the memory consumption and computation cost by using the above methods. In order to remove linear interferences from single images, transform techniques [28,29] and outlier detection methods [30,31] are often used. However, they only have a better effect on the interference conforming to linear distributions. The algorithm based on a variation of Laplacian edge detection [32] identifies cosmic rays of arbitrary shapes and size by the sharpness of their edges. Most of these algorithms have some defects and either do not correctly identify all the cosmic rays or sometimes identify faint objects. Therefore, they are not suitable for star image processing.

When strong interference exists in star images, a global threshold is impractical, and a set of adaptive thresholds based on local characteristics is required for the irregular background. Mao [33] proposed a local thresholding algorithm based on the Niblack algorithm. Arbabmir [9] also proposed a local threshold method for star image processing that is based on Bernsen algorithm. They can remove the uneven illumination interference but cannot remove it completely. A star is a type of small target in star images, and a top-hat transformation in mathematical morphology can be utilized in star detection and background estimation. Wei [34] proposed a method for the detection of small and weak targets by using a 1D morphology-based approach. Jiang [7] proposed a new star target segmentation(NSTS) algorithm based on a combination of three different structuring elements. They are available for specific interferences. However, the determination of the size of a structural operator is still a critical problem, especially for the complicated background.

To the best of our knowledge, in practical engineering applications, the star image is usually divided into several grids (such as 16×16) and the star point extraction algorithm is only implemented in the grids with little interference, which results in missing some effective star spots. For star identification, the recognition accuracy is related to the angular distance between two stars [35]. Therefore, it would be meaningful if the large-area interference region can be identified and removed before star extraction processing. It is a crucial premise to keep the star tracker working properly.

In recent years, the visual saliency model [36,37] has been widely used in the field of image segmentation and image retrieval because of its competence in simulating human visual characteristics and in intelligently extracting salient areas in images. In this paper, a new method based on SLIC-DBSCAN is proposed to remove large-area interferences in the star image. The star image is initially transformed based on luminance-based contrast (LC). Then, the simple linear iterative clustering (SLIC) algorithm is adopted in order to segment the saliency map by using the optimum parameters. The features are generated from these segments, and similar features are combined by using density-based spatial clustering of applications with noise (DBSCAN). A combination of these methods is adopted in order to accomplish the segmentation of the sky and interference region.

## 2. Background and Base

### 2.1. Description of Star Spot and Large-Area Interference

A typical star image consists of dozens of bright star spots with a dark background [38]. The signal-to-noise ratio (SNR) is generally between 20 dB and 50 dB. The energy of the star spot in the image plane can be considered as Gaussian diffusion [1]. The energy Gaussian distribution of the static star spots is defined in Equation (1).

(1) $$\begin{array}{cc} I(x,y) & =\int_{0}^{△t}f\left(x-x_{0},y-y_{0}\right)dt\\ \; & =\int_{0}^{△t}(\frac{E_{sum}^{M_{v}}}{2\pi \sigma_{PSF}^{2}}exp[-\frac{{\left(x-x_{0}\right)}^{2}}{2\sigma_{PSF}^{2}}]exp[-\frac{{\left(y-y_{0}\right)}^{2}}{2\sigma_{PSF}^{2}}])dt \end{array}$$

**Figure 1:**
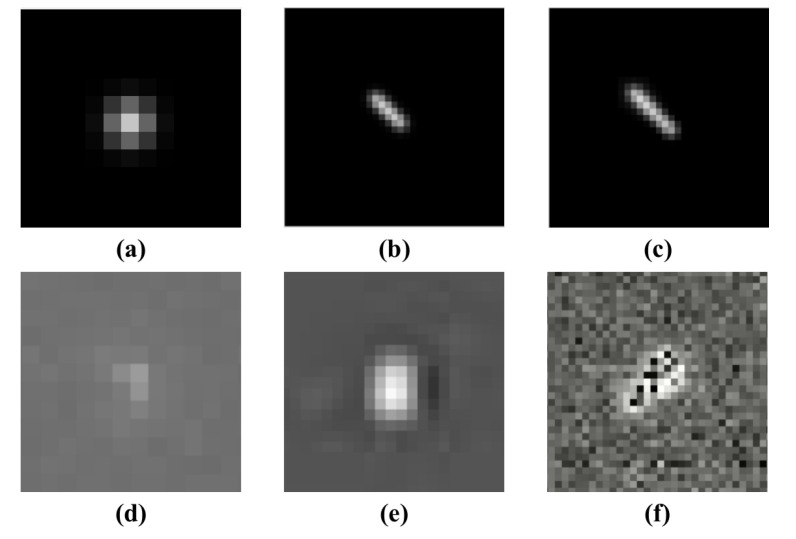
Schematic diagram of star spot energy distribution: (**a**) typical energy distribution of a star spot; (**b**) simulation of star spot under the condition of uniform motion; (**c**) under the condition of variable motion; (**d**–**f**) are the real energy distributions of the star spot in the real star images on orbit. There is a smearing phenomenon in (**f**).

The energy distribution model of star spots in dynamic case [39] can be expressed as Equation (2):

(2) $$I\left(x,y\right)=\int_{0}^{△t}f\left[x-x_{0}\left(t\right),y-y_{0}\left(t\right)\right]dt$$

where $\left(x_{0},y_{0}\right)$ represents the real center position of the star spot, and $\sigma_{PFS}$ is the Gaussian radius which represents the PSF of energy concentration. $E_{sum}^{M_{v}}$ is the energy-gray coefficient related to the apparent magnitude of the corresponding star, the quantum efficiency, the integral time, the lens aperture and the optical transmittance. In $t=t_{0}+△t$ (△t≪T, *T* represents the exposure time, and $\left(x_{0}\left(t\right),y_{0}\left(t\right)\right)$ is the centroid coordinates of the star spot at time *t*. The energy distribution of star spots is shown in Figure 1.

**Figure 2:**
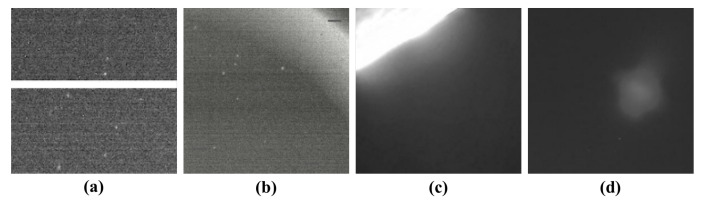
Characteristics of large-area interference: (**a**) linear interference; (**b**) design defects of light shield; (**c**) reflected light enters the star tracker; and (**d**) reflected light from celestial bodies enters the field of view.

This paper mainly focuses on analyzing three types of interferences as shown in Figure 2. Linear interferences are in Figure 2a. It produces linear stripes when light crosses the star image planes, including high-energy particles especially protons, satellites tracks, meteors or rapidly moving objects. This kind of interference may appear anywhere in the star image. Meanwhile, the length of the tracks is variable due to the relative velocity difference. Within the limited exposure time, the length of interference is significantly different from that of the typical star spot. With the rapid increase in satellites and the height of satellite orbit, space interferences are becoming more complicated, and such situations are becoming more common in the future. Regular-shaped interference in Figure 2b,c results in a rise in gray levels of the local pixels of star images. These interferences may be brought about due to the design flaw of the light shield, satellite components or their reflected light entering the field of view. Moreover, regular-shaped interference always appears around the star image. A special case is shown in Figure 2d. The irregular-shaped interference appears in the center of the star image, which results in the most fatal influence on the star tracker.

Compared with typical star spots, the interference is essentially different in shape and intensity. The large-area interference comprises contiguous blocks of pixels, and their gray values are near or higher than those of the star spots. For a general star image, the gray level contrast between the star spot and background noise is obvious. A global threshold with fast processing speed is sufficient. However, when large-area interference exits, an adaptive threshold is required for the irregular background. Traditional star segmentation method may not be a good choice for the varying background. The stars segmented from star images include many false star spots in the region of large-area interference. The algorithms implemented in FPGA cannot output the accurate attitude quaternion.

### 2.2. Luminance-Based Contrast Transformation

**Figure 3:**
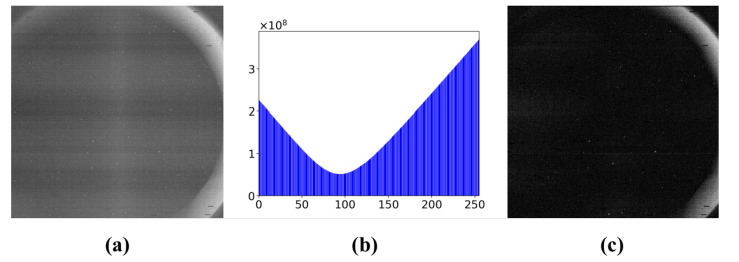
Saliency value calculation of star image: (**a**) star image with large-area interference; (**b**) the saliency values of the gray values; and (**c**) the resulting saliency map.

LC [40,41] is utilized in calculating the saliency values corresponding to the gray values of star image. It is a crucial step for better segmentation in order to increase the contrast of gray values. Specifically, the saliency value of a pixel in the star image is defined as its gray value contrast with all other pixels in the star image. The definition of LC is shown in Equation (3):

(3) $$Sal\left(I_{k}\right)=Sal\left(g_{s}\right)=\sum_{s=0}^{n}f_{s}\left\|I_{k}-g_{s}\right\|$$

where n=255 is the total numbers of gray level in the star image, and $g_{s}\in \left[0,255\right]$, $f_{s}$, is the frequency of pixel value $g_{s}$ in the star image. The ‖·‖ represents the distance metric (see also [40]). By using the saliency transformation of Equation (3), we obtain the saliency value corresponding to each gray value in the range of [0,255]. The result of LC is shown in Figure 3.

### 2.3. Simple Linear Iterative Clustering

SLIC [42] is used to segment the saliency map obtained from LC. The processing of SLIC algorithm is as follows:

1. Initialize parameter K, which is the desired number of approximately equally sized superpixels.
2. Move cluster centers to the lowest gradient position in a 3×3 neighborhood.
3. Calculate distance metric and assign the seed. The calculation of distance is as follows:
  (4) $$D=\sqrt{{d_{c}}^{2}+{(\frac{d_{s}}{S})}^{2}m^{2}}$$
  where *D* is the combination distances; $d_{c}$ and $d_{s}$ are color distance and space distance, respectively. $d_{s}$ and $d_{c}$ are computed as described in Equation (5).
  (5) $$\begin{array}{cc} d_{s} & =\sqrt{{\left(x_{j}-x_{i}\right)}^{2}+{\left(y_{j}-y_{i}\right)}^{2}}\\ d_{c} & =\sqrt{{\left(l_{j}-l_{i}\right)}^{2}+{\left(a_{j}-a_{i}\right)}^{2}+{\left(b_{j}-b_{i}\right)}^{2}} \end{array}$$
4. Compute residual error and iterate to make the error less than the setting threshold.

## 3. Extracting Sky Region

The traditional star image processing algorithm may not be working when the star image is interfered by large-area interference. The proposed algorithm mainly concentrates on addressing the large-area or strong interference issue. The main purpose of our algorithm is to divide the star image into two parts. One is the sky region; the other is interference region. Then, a star spot extraction algorithm is implemented only in the sky region. The interference region is marked and does nothing in this area. For this reason, the proposed algorithm is the reinforcement of traditional star image processing.

An intelligent algorithm for segmenting the sky and interference region in a single star image is proposed. The method is a combination of saliency detection, SLIC and DBSCAN. A constraint equation is added to LC. We named it as “restricted LC”, and it is utilized in the pre-processing of a star image. Then, SLIC is used to divide superpixels from the saliency map and to generate features from these superpixels. Finally, DBSCAN is utilized to cluster superpixels with similar features.

**Figure 4:**
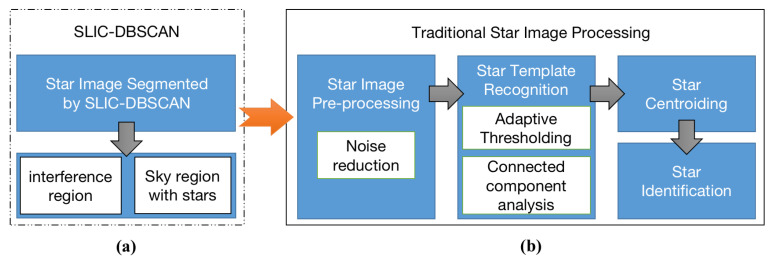
Proposed algorithm is the pre-processing for star images with large-area interference. They can be accomplished in two steps: (**a**) SLIC-DBSCAN is utilized to detect the region of large-area interference; (**b**) the other star spot extraction algorithm is implemented to segment stars, and the noise (such as single point noise, etc.) reduction technique is also considered.

**Figure 5:**
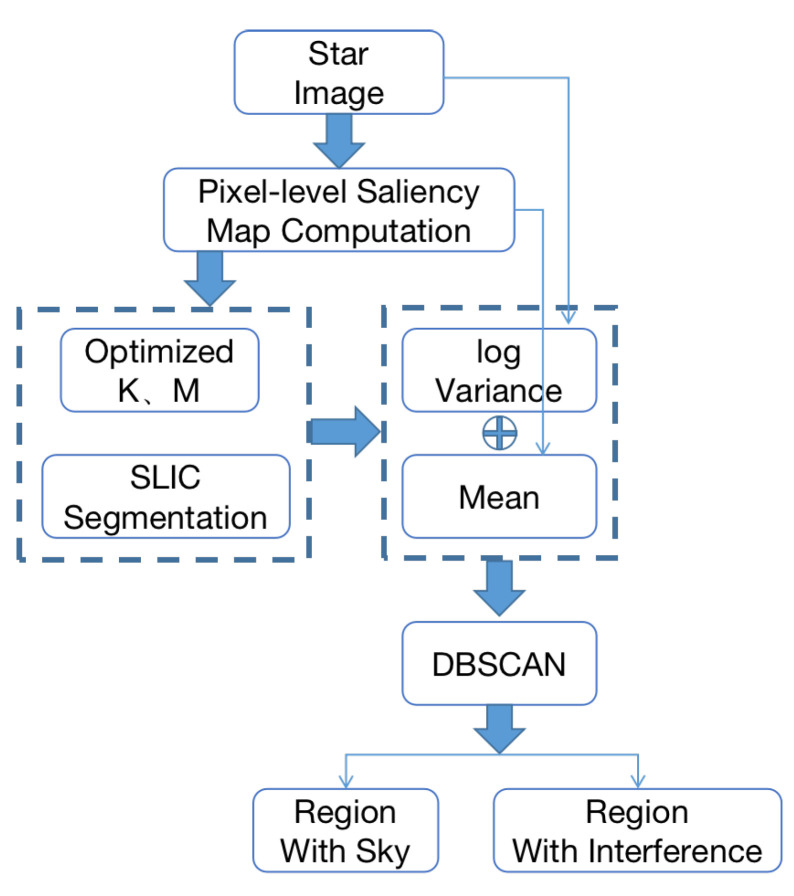
Flowchart of the proposed method.

The whole flowchart of the proposed and traditional algorithm is shown in Figure 4. The details of the proposed algorithm can be described in Figure 5.

### 3.1. Restricted LC

**Figure 6:**
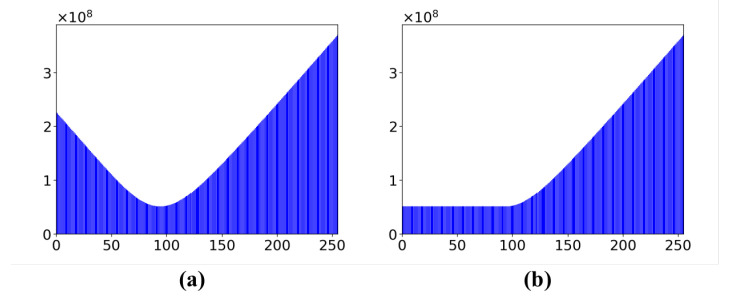
Comparison of LC and restricted LC: (**a**) the saliency values of the gray values using original LC algorithm; (**b**) the saliency values using restricted LC algorithm.

In Section 2, the gray level contrast gradients among the large-area interference, the star spots and the background noise are small; it is quite difficult to effectively separate them by simply setting thresholds or by computing the gradient of the star image. LC is adopted to increase the gray level contrast gradient among three parts of the star image. From Figure 3, it can be observed that the pixels with large gray values obtain larger saliency values after the LC transformation, while the saliency values of pixels with smaller gray values also become large. Dark pixels increase their proportion in the saliency map, which affects the segmentation result. In order to decrease the impact of dark pixels on the segmentation results, a constraint equation is added to the saliency value calculation, and it is renamed “Restricted LC”. The restricted LC is defined in Equation (6):

(6) $$Sal\left(I_{k}\right)=\left\{\begin{array}{cc} \sum_{s=0}^{n}f_{s}\left\|I_{k},g_{s}\right\| & I_{k}>=\underset{I}{\arg\min}Sal\left(I\right)\\ \sum_{s=0}^{n}f_{s}\left\|I_{m},g_{s}\right\| & otherwise \end{array}\right.$$

where $I_{m}=\underset{I}{\arg\min}Sal\left(I\right)$ represents the minimum value of saliency values. The details of restricted LC are shown in Figure 6. Background pixels make up the majority of the star image, and their gray values are usually in the range of [0,130]. After the calculation of restricted LC, pixels with large gray values still obtain higher saliency values, but the pixels of the background with small gray values obtain small saliency values. In other words, the star spot and the interference obtain large saliency values, and the background pixels possess small saliency values.

### 3.2. Optimum Parameters in SLIC

SLIC is utilized in order to segment the saliency map obtained from Section 3.1. SLIC can segment the star image into semantically meaningful sub-regions such as sky regions or interference regions. The superpixels segmented by SLIC adhere well to image boundaries, and the local features of the star images can be described by superpixels instead of pixels of the star image. Compared to grid segmentation, SLIC is easier in application for segmenting the regions into irregular shapes; the interference region of star image is usually irregular-shaped.

Instead of computing distance $d_{c}$ in Equation (5), we chose saliency values $Sal(I_{k})$ and space coordinates (x,y) as the cluster centers $G={\left[Sal,x,y\right]}^{T}$. The distance between saliency values $d_{sal}$ is defined by Equation (7).

(7) $$d_{sal}=\sqrt{{\left({Sal}_{j}-{Sal}_{i}\right)}^{2}}$$

In order to combine the $d_{s}$ and $d_{sal}$ into a single measure $D^{\prime }$, Equation (8) is used:

(8) $$D^{\prime }=\sqrt{{d_{sal}}^{2}+{(\frac{d_{s}}{S})}^{2}m^{2}}$$

where $S=\sqrt{\left(N/K\right)}$ is the initial grid interval, *i* is the clustering center and *j* is pixels near the clustering center. The expected spatial extent of a superpixel is a region of approximate size S×S. The search for similar pixels is performed in a region 2S×2S. $Sal(I_{k})$ is normalized into [0,255].

The parameter *m* is a normal scalar, which is used to balance the saliency distance and the spatial distance in the distance metric. When *m* is large, the spatial proximity is more important, and the resulting of superpixels is more compact (i.e., they have a lower area to perimeter ratio). When *m* is small, the resulting superpixels adhere more tightly to image boundaries, but have irregular size and shape.

**Figure 7:**
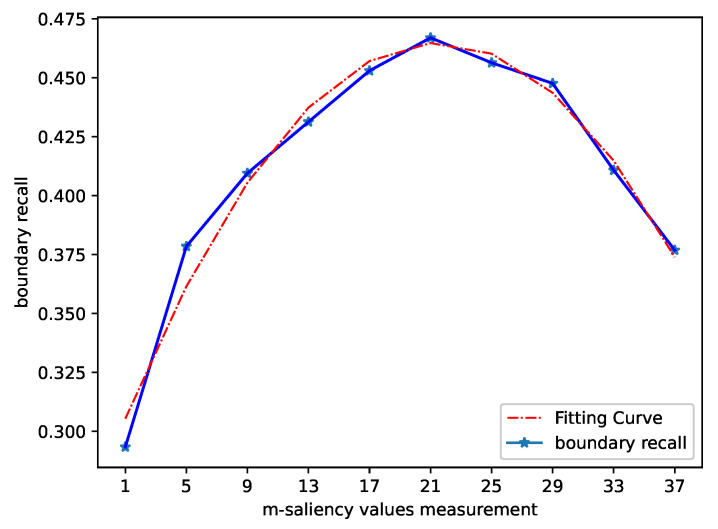
The boundary recall statistics of different *m*; the red curve is the fitting result of boundary recall.

In order to synthesize a segmentation that adheres more to the boundaries and also to split out the interference region as completely as possible, we perform statistical analysis of the segmentation results of different *m* and use boundary recalls [43] to measure its ability to adhere to image boundaries. Boundary recall measures the fraction of the ground truth edges fall within at least two pixels of a superpixel boundary. The boundary recall of each *m* is plotted in Figure 7 for increasing numbers of *m*. A high boundary recall indicates that very few true edges were missed. From the statistics of boundary recall, the evaluation of *m* should be 21.

**Figure 8:**
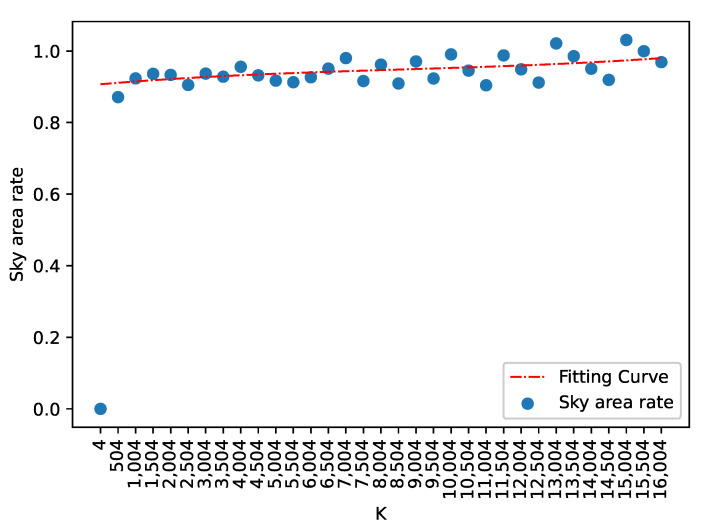
The sky occupancy statistics of different *K*; the red curve is the fitting result.

The initial grid interval is $S=\sqrt{\left(N/K\right)}$, while the parameter K has different values in different image classification tasks. The sky occupancy is defined as a ratio of the sky region to the star image:

(9) $$A_{sky}\left(K\right)=\frac{\sum_{i=1}^{K}\left(A_{i}\in C_{sky}\right)}{K}$$

where *K* is the number of superpixels, $A_{i}$ is the *i*th superpixel and $C_{sky}$ represents the superpixel of the sky region after clustering in Section 3.3. Using SLIC in star image, if *K* is too large, the superpixel may only contain a star spot, and the star spot is removed as interference occurs in the subsequent processing. If *K* is too small, the segmentation will be not accurate, and the superpixels may not adhere well to image boundaries. The sky occupancy ratio of different *K* is plotted in Figure 8. It can be observed that $A_{sky}^{K}$ becomes divergent for increasing numbers of *K*, indicating that the superpixel is over-segmentation. *K* is closely related to the size of the image, and we recommend that *K* should be chosen in the range of [1,2] times the length of the star image.

### 3.3. Extracting Features and DBSCAN

The difference between star spot and interference is that star spot principally exists with a Gaussian distribution within a window between 3 pixels × 3 pixels and 5 pixels × 5 pixels [1]. While the interferences occupy more pixels, they are usually contiguous pixel blocks and do not possess fixed shapes. The superpixels obtained from Section 3.2 contain star spots or large-area interferences.

**Figure 9:**
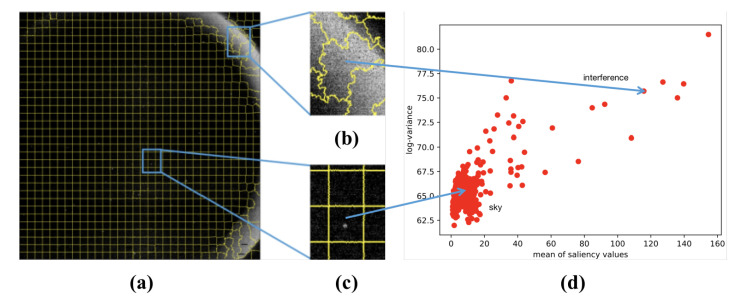
Feature extraction: (**a**) the result of optimized SLIC segmentation; (**b**) details of large-area interference region; (**c**) details of region with suspected star spot; and (**d**) distribution of feature vector extracting from superpixels.

The human visual system is sensitive to information such as the boundary of the scene, the variance distribution of spatial pixels, the discrepancy of gray values and boundary fitting [44]. Based on the characteristics of the visual system and the interference analysis in Section 2.1, we extract a feature vector from each superpixel as shown in Equation (10):

(10) $$S_{i}=\left[M_{i},V_{i}\right]$$

where $M_{i}$ is the mean of saliency values of the *i*-th superpixel, and $V_{i}$ is the variance of gray values of original star image in *i*-th superpixel. The schematic diagram of feature extraction is shown in Figure 9. It can be observed that the feature vector $S_{i}$ of the sky region superpixels is close to one another, and in the large-area interference region $S_{i}$ is divergent and far apart from one another. In other words, the sky region has similar local features while the large-area interference region does not have such characteristics. There is a distinct difference between the density characteristics of samples.

DBSCAN [45] is utilized to cluster superpixels with similar features. DBSCAN assumes that the sample category can be determined by the tightness of the sample distribution. Points that are closely packed can be grouped together, while points that lie alone in low-density region are marked as outliers. The features of the sky region are similar, and the their distribution is concentrated. Consequently, these features can be clustered. However, the features of large-area interference region are quite different, and the distribution is not concentrated; thus, theywould be marked as outlier points in DBSCAN. The computational complexity of DBSCAN is related to the dataset dimension, and it is not suitable for high dimensional datasets. The features $S_{i}$ of the proposed method is 2. Compared to other clustering algorithm, DBSCAN requires less computation and is particularly suitable for density clustering.

The core of DBSCAN is to calculate the distance between samples to find all core objects. The distance metric is Minkowski distance:

(11) $$D_{sp}=\sqrt{\sum_{p=1}^{q}{\left(|S_{i}^{p}-S_{j}^{p}|\right)}^{2}}$$

where $S^{p}$ is the feature vector extracted from the superpixels, and *q* is the dimension of the feature vector. In the proposed method, q=2. The mean $M_{i}$ of saliency values and the variance $V_{i}$ of gray values in each superpixel have different weights in the distance measurement. In the superpixel of large-area interference, the variance takes a larger weight in the distance measurement. The superpixels of the sky region are changed slightly, and their variance remains greater than the mean. Therefore, in the distance measurement, we used $ln(V_{i})$ instead of $V_{i}$ to weaken the weight of variance. The weighted Minkowski distance is expressed as follows.

(12) $$D_{sp}=\sqrt{{\left(|M_{i}-M_{j}|\right)}^{2}+(|ln\left(V_{i}\right)-ln\left(V_{j}\right){\left|\right)}^{2}}$$

A cluster set $C=\left\{C_{0},C_{1},\cdots ,C_{k}\right\}$ can be obtained from DBSCAN, where the clusters satisfying the characteristics of the sky region are the effective sky regions extracted from a single star image with large-area interference.

### 3.4. Complexity

The proposed algorithm consists of three parts, meaning that the proposed method is a summation of three complexities. The computational complexity of both restricted LC and SLIC is O(N). The average-case complexity of DBSCAN is O(nlog(n)), and the worst-case complexity of DBSCAN is $O(n^{2})$. Therefore, the total time computational complexity of the proposed method is O(N+nlog(n)).

### 3.5. Pseudocode

We summarize our methods for extracting effective sky region of star images in Algorithm 1.
**Algorithm 1:** Sky Region Extraction Using a SLIC-DBSCAN based algorithm.
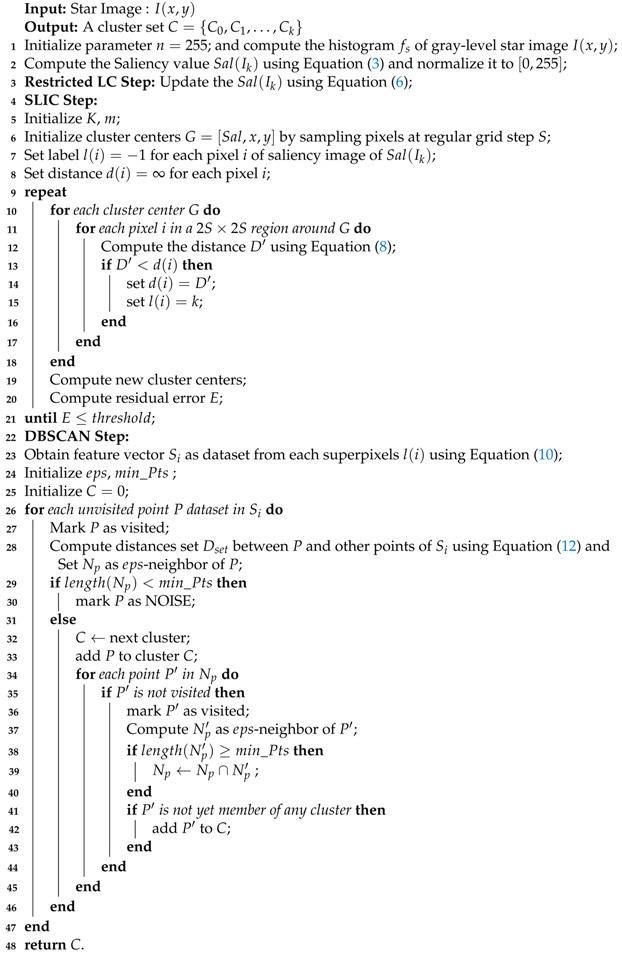


## 4. Experiments and Results

All experiments were conducted for real star images on software platforms in order to verify the superiority of our method. All algorithms were simulated in PyCharm CE(V2020.2) with Python 3.7 in a PC with a 2.3 GHz Core i5 CPU, 8 GB RAM and a Macintosh operational system. The computation time for a given 1024 × 1024 real star image is 8.74183 s.

### 4.1. Comparison with Different Clustering Algorithms

**Figure 10:**
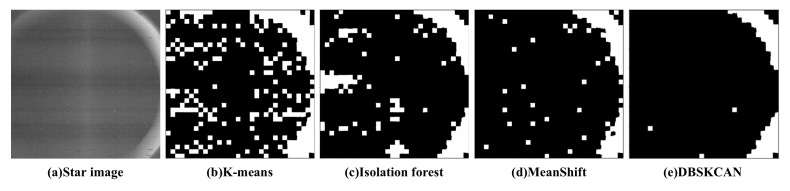
(**a**) The star image with strong interference and result of (**b**) K-means [46]; (**c**) Isolation forest [47]; (**d**) Meanshift [48]; and (**e**) DBSCAN [45]. In the binary image, white pixels are the region of large-area interference. The black pixels are the region of the sky.

In order to justify that DBSCAN produces better clustering in the proposed algorithm. K-means [46], isolation forest [47] and meanshift [48] were used in the experiment for a simple comparison with DBSCAN. The results are shown in Figure 10. It shows that a more complete sky region can be segmented by DBSCAN.

### 4.2. Comparison with Existing Stray Light Suppression Algorithms

**Figure 11:**
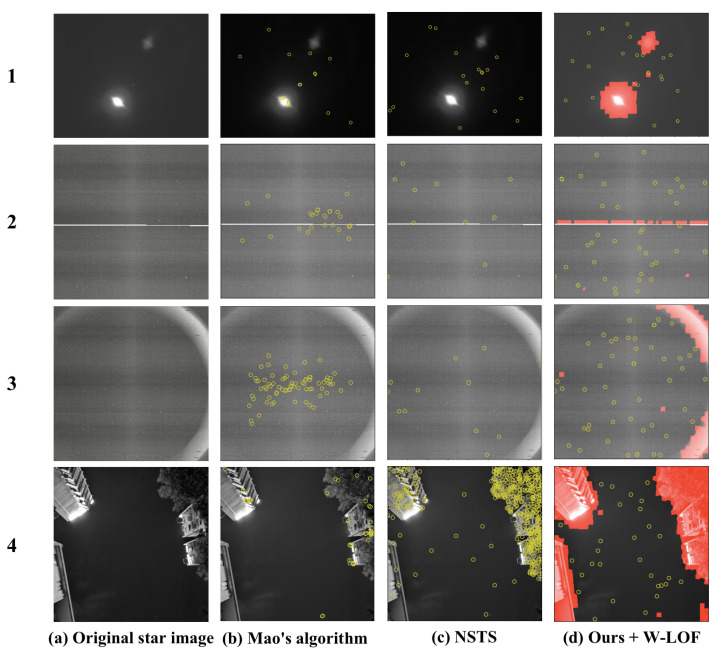
Result of star spot extraction with different algorithm: (**a**) original star image with different interferences; (**b**) potential stars using Mao’s algorithm; (**c**) potential stars using NSTS; and (**d**) potential stars with our method and Wavelet-LOF algorithm.

Arbabmir’s algorithm [9], Mao’s algorithm [33] and NSTS [7] were applied to several star images with different stray lights and varying illumination such as moonlight. Given that Arbabmir’s algorithm cannot remove the interference in a star image, Mao’s algorithm and NSTS were compared with our algorithm. In particular, these algorithms were implemented directly on the star images, and the potential stars were extracted. While our method is primarily used to identify the large-area interferences in the star images and to remove them, the star spot extraction algorithm is utilized to segment stars in the region without strong interferences. Figure 11a shows the original star image with different interferences; from the top to the bottom are star images with moonlight, linear interference and interferences with irregular shapes. Figure 11b shows the potential stars using Mao’s algorithm. Figure 11c shows the potential star spots using NSTS, and Figure 11d is the result of using our method with W-LOF.

The experimental results show that Mao’s algorithm and NSTS have the advantage of suppressing large-area interference such as moonlight. However, they do not perform well in suppressing complex interferences such as annulus interference; they either extracted a number of false star spots or cannot obtain enough star spots for star identification. The proposed algorithm can suppress various categories of interferences and is not sensitive to the shape of the interference. The availability of star images can be improved.

#### 4.2.1. Ratio of Available Stars to Extracted Stars

**Figure 12:**
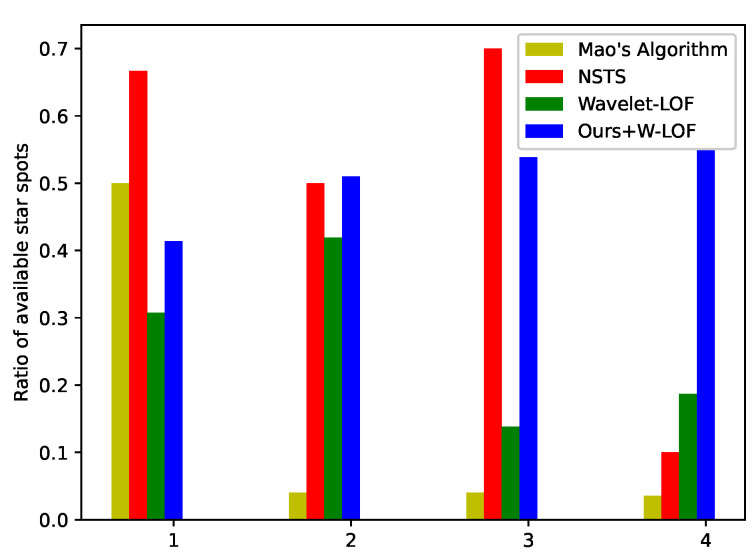
Comparison of the ratio of available star spots. One to four are star images in Figure 11 (1–4).

Mao’s algorithm, NSTS and W-LOF were compared with ours to verify the validity of the latter. Four images in Figure 11a were processed by Mao’s algorithm, NSTS, W-LOF and the proposed method with W-LOF. The ratio of available stars to total number of segmented stars was calculated. As shown in Figure 12, Mao’s algorithm and W-LOF have shortcomings in dealing with star images with large-area interferences. Although NSTS has high ratio of available stars, the number of stars segmented by NSTS is less than others. After removing the large-area interference with the proposed method, almost all the false star spots can be eliminated, and the proportion of available star spots has increased significantly.

#### 4.2.2. Probability of True Detection and Miss Detection

**Figure 13:**
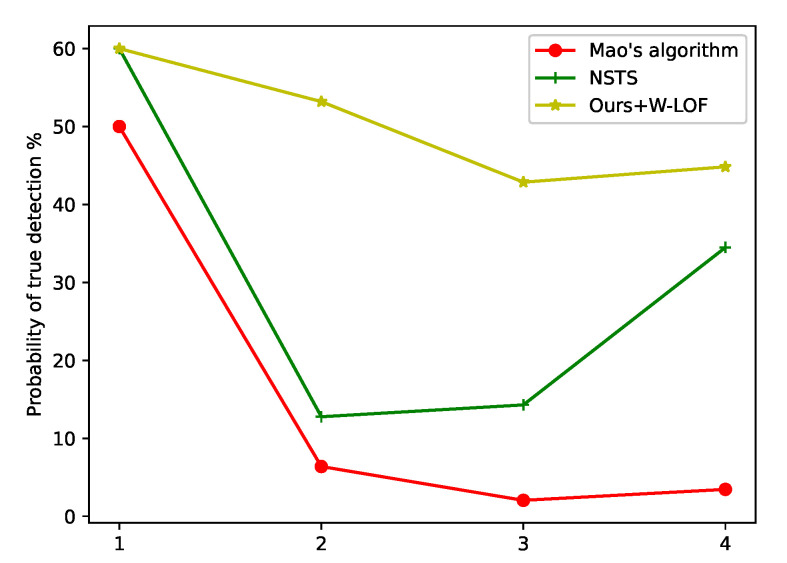
Comparison of the probability of the true detection. One to four are star images in Figure 11 (1–4).

**Figure 14:**
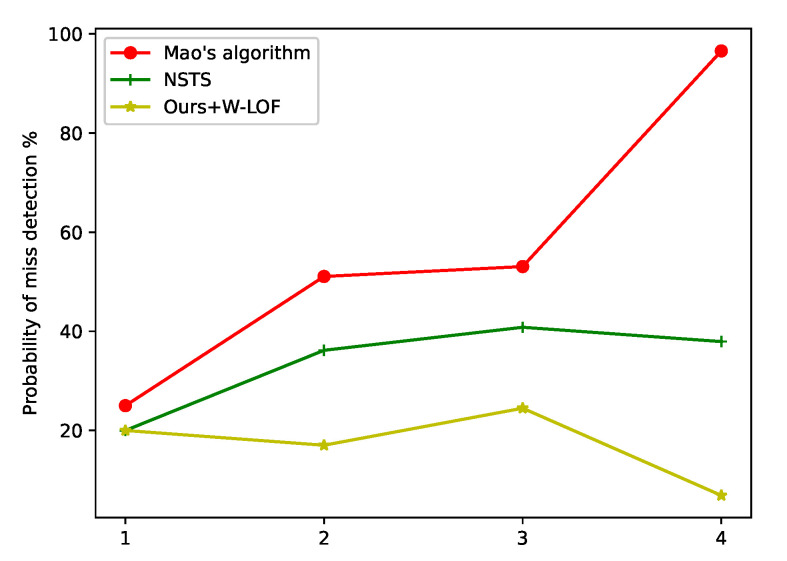
Comparison of the probability of the miss detection. One to four are star images in Figure 11 (1–4).

In order to validate the improvement of the proposed method, we compared the probability of true detection and miss detection of Mao’s algorithm, NSTS and the proposed method with W-LOF. After using these methods to segment star spots, star identification [49] was performed by the same method, and the probabilities of true detection and miss detection were calculated, as shown in Figure 13 and Figure 14. On account of the large-area interference in the star image, star identification cannot be evaluated using Mao’s algorithm and NSTS. Our method primarily removes the region of interference, and most false stars in the region of interference are eliminated. The probability of true detection significantly increased by using the proposed method. After implementing the proposed method and removing the large-area interference, the star extraction algorithm is not disturbed by false stars; as a result, the probability of miss detection is lower than others algorithms.

### 4.3. Star Image in the Real Night Sky Observation Experiments

**Figure 15:**
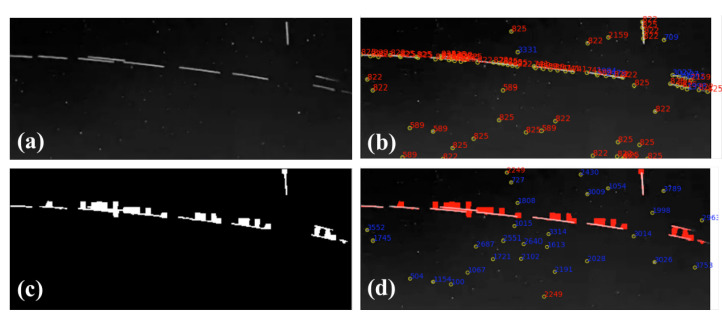
Linear interference by Starlink satellite: (**a**) the star image from Astronomical Observatory CCAF; (**b**) the extraction result with the Wavelet-LOF algorithm; (**c**) binary mask generated by proposed method; and (**d**) the result of star spot extraction with the mask. The red points indicate incorrect recognition, and the blue points indicate correct recognition.

The star image of the real night sky observation experiments was tested. The star image interfered by the Starlink constellation satellites is shown in Figure 15. In Figure 15a, a set of satellites pass through the field of view of star tracker, causing a linear interference. The frequently used threshold segmentation algorithm does not work because many false star spots are extracted. After identifying the large-area interference regions and covering them with masks (Figure 15c) using the proposed method, the star spots can be extracted successfully in the sky region by W-LOF or other star extraction algorithms.

**Figure 16:**
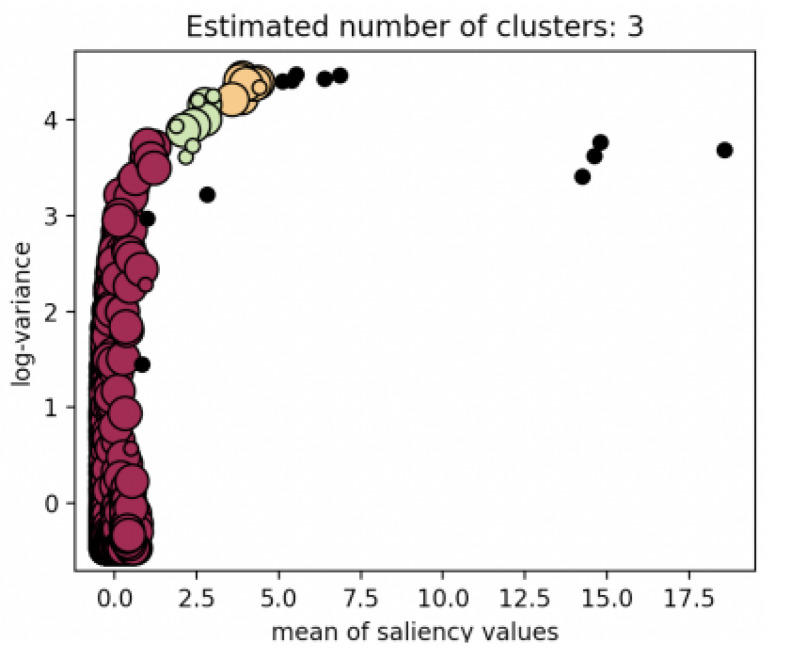
Clustering result by DBSCAN; the red cluster represents the sky region, the clusters with other color are interference region.

The parameter radius epd is set to 0.4 and min_Pts=5 in DBSCAN, which are selected by statistical result. In Figure 16, the red points are the clusters of sky region, and the other colored points are treated as noise. We combine the superpixels labeled as noise, and the results are shown in Figure 15c. To prove that the proposed algorithm is effective, we extracted star spots using W-LOF. The star spot extraction algorithm was only implemented within the sky region. The results of star spots extraction are shown in Figure 15d.

**Figure 17:**
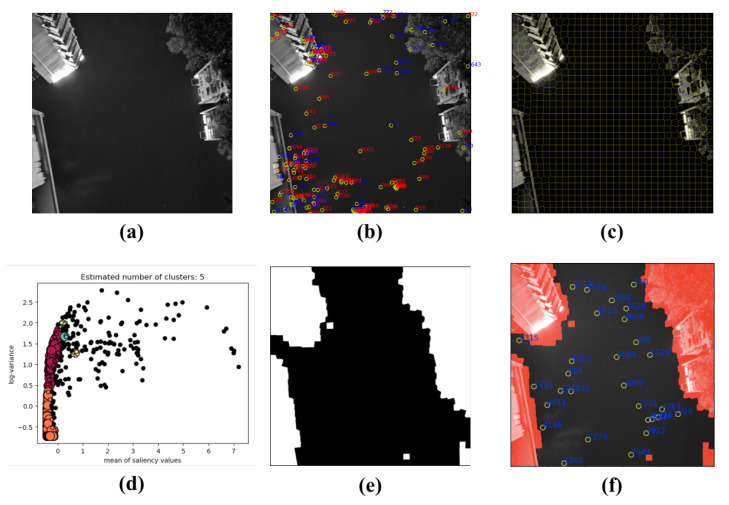
(**a**) The star image; (**b**) extraction results with the Wavelet-LOF algorithm (there are many false star spots); (**c**) result of SLIC segmentation; (**d**) result of DBSCAN clustering; (**e**) binary mask generated by proposed method; and (**f**) result of star spots extraction with the mask. The red points indicate incorrect recognition, and the blue points indicate correct recognition.

The experimental result of another situation with complex interference is shown in Figure 17. Strictly speaking, this situation usually does not happen with satellites. We take this situation into consideration because, under the requirement of low-cast and high-performance of future star trackers, the real night sky observation experiments conducted in cities become possible. The situation is more complex than others, and it can verify the capability of the proposed algorithm.

### 4.4. Star Image from On-Orbit Satellites

**Figure 18:**
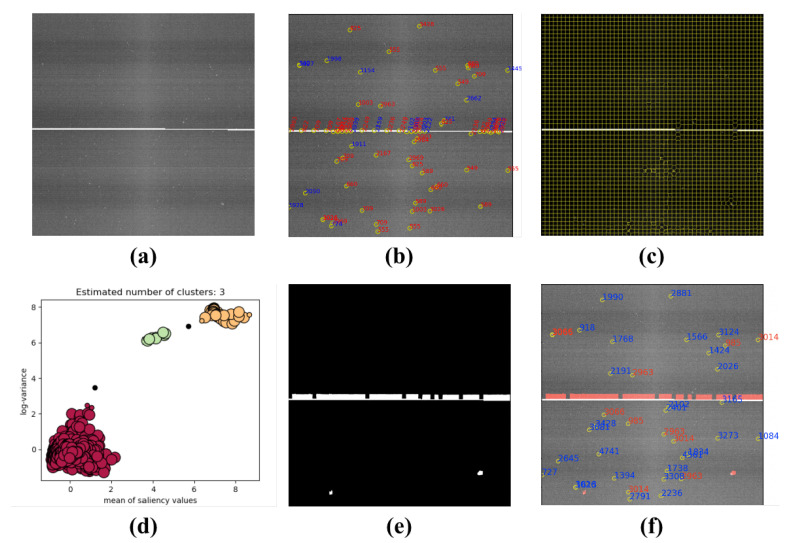
(**a**) The star image with linear interference; (**b**) the extraction result with W-LOF, there are many false star spots; (**c**) the result of SLIC segmentation; (**d**) the result of DBSCAN clustering; (**e**) binary mask generated by proposed method; and (**f**) the result of star spots extraction only in the sky region. The red points indicate incorrect recognition, and the blue points indicate correct recognition.

**Figure 19:**
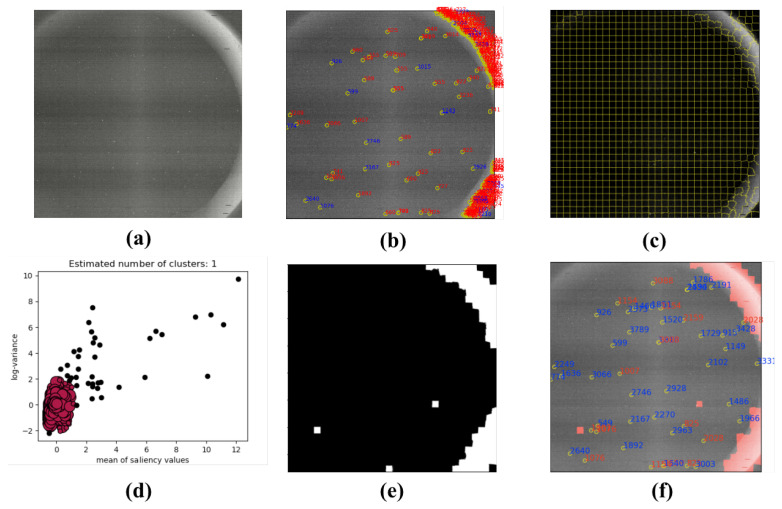
Regular annulus interference: (**a**) the star image with regular annulus interference; (**b**) the extraction result with W-LOF (there are many false star spots); (**c**) the result of SLIC segmentation; (**d**) the result of DBSCAN clustering; (**e**) binary mask generated by proposed method; and (**f**) the result of star spot extraction only in the sky region.

**Figure 20:**
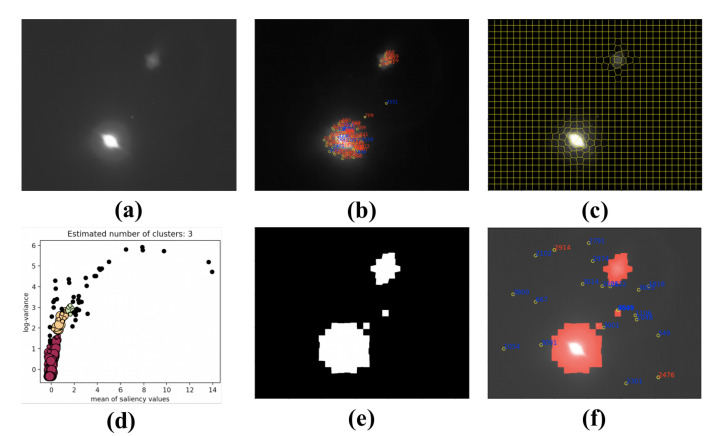
Irregular shaped interference with moonlight in the star tracker: (**a**) the star image with moonlight; (**b**) the extraction result with W-LOF algorithm (there are many false star spots); (**c**) the result of SLIC segmentation; (**d**) the result of DBSCAN clustering; (**e**) binary mask generated by proposed method; and (**f**) the result of star spot extraction only in the sky region.

Figure 18a, Figure 19a and Figure 20a are the star images from on-orbit satellites. Figure 18 displays the linear interference, and Figure 19 is regular annulus interference caused by the design defects of the star tracker shield. They came from the same star tracker of the on-orbit satellite. Figure 20 shows the irregular shaped interference caused by the moonlight entering the field of view of the star tracker. From the results, it can been observed that the features of on-orbit star image are obvious, and they have a clear boundary to segment the sky region and interference region.

## 5. Conclusions

With the upsurge of satellites, the star trackers are crucial for attitude determination. Since the star image is the only data source for the star tracker, the presence of large-area interference is fatal for its performance. In the worst case scenario, the star tracker cannot work properly.

In order to ensure the safety of the spacecraft when the star tracker breaks down, the method based on SLIC-DBSCAN is proposed for segmenting the sky and interference region in a single star image. It integrates saliency detection, SLIC and DBSCAN. By using the proposed algorithm, a star image is divided into two parts: One is the sky region which is used to implement star image processing algorithm and to compute the accurate attitude; the other is the interference region.The algorithm of this study is devoted to solving the problems of the large-area interference. The experimental results showed that the proposed algorithm possesses good capability in suppressing large-area interference. It can also improve the proportion of available star spots. The experiments on star images from real night sky observation and on-orbit observations reveal that the proposed algorithm can identify various types of interferences and is not sensitive to the shape of the interference.

In the future, we plan to improve the method and to study large-area interference suppression under dynamic conditions. Furthermore, we will consider optimizing the proposed algorithm and implementing it relative to high-performance co-processors such as the LoongSon chip. The method proposed in this paper can be extended to the image recognition of satellite-carried optical sensors and the real night observation experiments.

## Data Availability

Not applicable.
